# Improving Pathways to Care for Patients at High Psychosis Risk in Switzerland: PsyYoung Study Protocol

**DOI:** 10.3390/jcm12144642

**Published:** 2023-07-12

**Authors:** Caroline Conchon, Elodie Sprüngli-Toffel, Luis Alameda, Anne Edan, Barbara Bailey, Alessandra Solida, Kerstin Jessica Plessen, Philippe Conus, Afroditi Kapsaridi, Davina Genoud, Aureliano Crameri, Sondes Jouabli, Camille Caron, Carmina Grob, Julia Gros, Smeralda Senn, Logos Curtis, Ana Liso Navarro, Remy Barbe, Nathalie Nanzer, Evelyn Herbrecht, Christian G. Huber, Nadia Micali, Marco Armando, Stefan Borgwardt, Christina Andreou

**Affiliations:** 1 General Psychiatry Service, Treatment and Early Intervention in Psychosis Program (TIPP–Lausanne), Lausanne University Hospital and University of Lausanne, 1003 Lausanne, Switzerland; 2 Psychology and Neuroscience, King’s College of London, London SE5 8AF, UK; 3 Centro Investigacion Biomedica en Red de Salud Mental (CIBERSAM), Instituto de Biomedicina de Sevilla (IBIS), Hospital Universitario Virgen del Rocio, Departamento de Psiquiatria, Universidad de Sevilla, 41013 Sevilla, Spain; 4 Child and Adolescent Psychiatric Service, Geneva University Hospital, 1211 Geneva, Switzerland; 5 Universitäre Psychiatrische Kliniken (UPK) Basel, Universität Basel, 4002 Basel, Switzerland; 6 Center of Psychiatry of Neuchâtel (CNP), 2000 Neuchâtel, Switzerland; 7 Division of Child and Adolescent, Lausanne Hospital (CHUV), 1004 Lausanne, Switzerland; 8 Faculty Biology and Medicine, University of Lausanne, 1005 Lausanne, Switzerland; 9 Zürcher Hochschule für Angewandte Wissenschaften (ZHAW), 8005 Zurich, Switzerland; 10 Young Adult Psychiatric Unit, Department of Psychiatry, Geneva University Hospitals, 1202 Geneva, Switzerland; 11 Medical Pedagogical Office, Department of Instruction, State of Geneva, 1200 Geneva, Switzerland; 12 Translational Psychiatry Unit, Department of Psychiatry and Psychotherapy, University of Lübeck, 23562 Lübeck, Germany

**Keywords:** early detection, early intervention, protocol, pathway to care, ARMS, FEP

## Abstract

Aims: Psychotic disorders are one of the main causes of chronic disability in young people. An at-risk mental state (ARMS) is represented by subclinical symptoms that precede the first episode of psychosis (FEP). The PsyYoung project aims to optimize the detection of an ARMS while reducing unnecessary psychiatric treatments. It investigates the effects of service changes on the referrals and outcomes of young people with ARMS or a FEP. Methods: Six psychiatric outpatient clinics in three cantons (Basel-Stadt, Vaud, and Geneva) participated in the project. They passed through an implementation phase including service changes and the adaptation of a standardized stepped care model for diagnosis and assessment, in addition to measures for increasing the awareness, networking and training of local professionals. Preliminary results: All participating cantons had entered the implementation phase. By March 2023, there were 619 referrals to participating sites. A total of 163 patients (37% FEP and 31% ARMS) and 15 close relatives had participated in individual longitudinal assessments, and 26 patients participated in qualitative interviews. Conclusion: This national collaborative project addresses the issue of early intervention for emerging psychoses, and creates spaces for fruitful reflections and collaboration in Switzerland. The ultimate aim of PsyYoung is to harmonize clinical practices in early intervention of psychosis on a national level.

## 1. Introduction

In Switzerland, approximately 25% of new disability pensions for people aged 25–29 are for health reasons related to schizophrenia or other psychotic disorders (PD) [1]. The OFS report also demonstrates that approximately 5/1000 men and 1.5/1000 women aged between 19 and 34 years old were hospitalized for schizophrenia spectrum disorder in 2018 [2]. Moreover, around 40% of patients with PD progress favorably in terms of positive symptomatology, but continue to show impairment in social and occupational functioning despite effective pharmacological treatment for positive psychotic symptoms. Meta-analytic data have shown that only around one in seven patients with a diagnosis of schizophrenia or schizophrenia spectrum disorder will recover from their initial episode [3], both in terms of symptoms and functioning. This highlights the need to improve the identification of patients at risk of developing a psychotic disorder, and to intervene early for those with already manifested disorders, in order to avoid a poor prognosis.

It has been established over the years that, in their early stages, psychotic disorders can be effectively treated, and that a shorter duration of untreated psychosis is associated with a better prognosis in terms of functioning and lower social consequences [4]. For these reasons, early intervention services (EIS) for psychosis have been developed since the mid-1990s [5], often with a focus on early treatment following a first psychotic episode (FEP), but also focusing on the so-called “prodromal” phases. The latter term corresponds to subclinical symptoms that exist before the onset of overt psychotic symptoms and can be detected by means of specific tools [6]. Currently, the prodromal phase is conceptualized around the notion of an “at-risk mental state” (ARMS) [7]. ARMS criteria are assessed by means of structured interviews such as SIPS (structured interview of prodromal syndromes [8]), CAARMS (comprehensive assessment of at-risk mental states [7,9]), or recently, Psysch [10]. An ARMS is defined generally by the following categories: (i) attenuated positive psychotic symptoms (APS); (ii) brief limited intermittent psychotic symptoms (BLIPS); and (iii) either a genetic risk or schizotypal disorder with functional decline (DRG) [5]. EIS are defined as a multidisciplinary community team providing treatment for individuals presenting with an ARMS or FEP [11]. There are various modalities, but the most common is the stand-alone model, which works independently from other more generic community teams [6]. The EIS model for FEP patients is based on the proactive intervention of the “case manager” (or “care coordinator)” (usually a nurse or social worker), who remains the main carer through the treatment period and who co-works with the medical doctors. The case manager is required to intervene early after the referral is made (usually a first contact with the patient needs to happen within the first 14 days after the referral [11]). EIS usually offer an integrated biopsychosocial treatment based on psychotherapy, psychoeducation, family support, cognitive assessment and remediation (when needed), social support, assistance in finding employment, and pharmacological treatment. The last is applied based on international guidelines and favors atypical antipsychotic treatment [12]. The case load of an EIS case manager is around 25–30, as compared with 70–80 for more traditional settings. This allows more flexibility and proactivity as well as intensive crisis management and assertive outreach, which is believed to be required in the early phases of psychotic disease [13]. While the EIS model is well defined for FEP patients, it is less clear how this model needs to be adapted for a population with an ARMS [6].

Meta-analytic data show that approximately 36–37% of patients who meet the criteria for an ARMS make a transition to psychosis within the first 2 to 3 years [14]. Although this represents a minority of patients in absolute terms, approximately one third of patients who do not transition to a PD present attenuated positive psychotic symptoms over the long term. Furthermore, the majority of ARMS patients develop another psychiatric pathology [15]; 60% of children and adolescents with ARMS present negative symptoms [16] and a level of functioning that is almost as low as that of patients with an established psychotic disorder [17]. This shows the need to not only identify these individuals with the aim to prevent a transition to a PD, but also to address the multiple clinical needs that they suffer from, regardless of psychosis risk. Furthermore, these individuals should be offered treatment that, in accordance with the guidelines, does not involve antipsychotic medication as a first-line treatment [18].

Currently in Switzerland, several cantons have set up EIS that use various approaches to serve the evidence-based, early identification and treatment of FEP and ARMS patients (Solida, A., et al. under review). However, one of the challenges of early intervention in psychosis is to identify individuals with an ARMS early on, before they develop enduring impairment of functioning or a full-blown psychotic disorder. This involves improving the pathway to care system by informing and educating primary networks regarding these types of manifestations, and encouraging early referral of suspected patients with an ARMS to an early intervention team [6]. Another major challenge is to reduce DUP in FEP patients, which is a major goal of EI services and remains a major determinant of a poor outcome [4].

The PsyYoung project aimed to address the above challenges. The project started in 2019, funded by Health Promotion Switzerland (Promotion Santé Suisse), within the framework of the incentive program “Prevention in Healthcare”. This was initiated in collaboration with the Federal Office of Public Health to support the national strategy for the prevention of non-communicable diseases [19]. PsyYoung started in December 2019 and will end in March 2025.

### Aims and Hypotheses

PsyYoung aims to investigate the effects of complex service delivery changes on the referrals and outcomes of adolescents and young adults with an ARMS for psychosis. The service delivery changes consist of a standardized stepped-care model for access to early intervention, in combination with awareness and networking activities targeting professionals in the education and healthcare sectors. These changes (henceforth referred to as an ‘intervention’) will be implemented in three cantons (Basel-Stadt, Vaud, Geneva), including six EIS from the departments of adult, and child and adolescent psychiatry of three university hospitals (CHUV: University Hospital of Lausanne; HUG: University Hospitals of Geneva; UPK: University Psychiatric Clinics Basel) and the Department of Public Education (DIP) of the Canton of Geneva.

It is expected that the PsyYoung intervention will (i) optimize pathways to care and facilitate the timely referral of young people with a potential ARMS and FEP to specialized early intervention sites; (ii) improve clinical outcomes, quality of life, and functioning in these patients a year after referral; and (iii) lead to a reduction in unnecessary psychiatric treatments, particularly treatment with antipsychotics, in patients with a low psychosis risk. 

## 2. Methods

### 2.1. Intervention: Service Delivery Changes

#### Standardized Stepped-Care Diagnosis and Assessment Model

**Figure 1:**
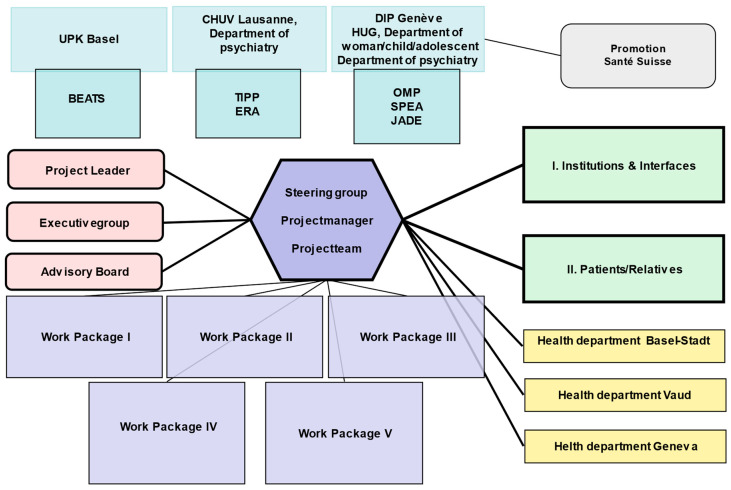
PsyYoung sites (in blue; BEATS—Basel early treatment service, TIPP—treatment and early intervention program for psychotic disorders, ERA—psychotic risk assessment, OMP—medico-pedagogical office, SPEA—child and adolescent psychiatry service, JADE—young adults with early onset disorders program), project structure (shades of purple), and relationships with the funding institution (grey) and external partners. WP (work package) 1 is responsible for developing the consensus standard on referral and orientation procedures, based on documentation of the current practices. WP2 established the consensus m assessment with recommendations for diagnostic instruments and procedures, which are prioritized based on their level of usefulness; the same WP is responsible for developing training materials. WP3 is responsible for the digital infrastructure of the project, including the website and the risk calculator. WP4 manages data collection and management, quality management, and ethics; and finally, WP5 focuses on communications, networking, and sustainability, including the development of new financing models for early intervention.

**Figure 2:**
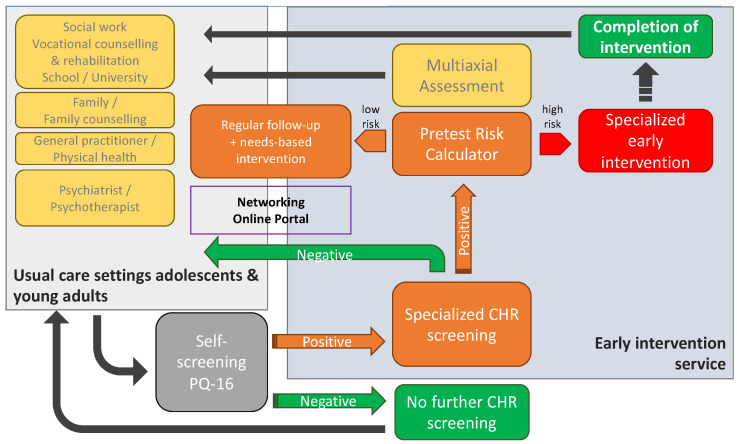
Implementation phase. If the PQ-16 is positive, patients can be addressed in the EIS and will be screened for the CHR-P criteria by the EIS; patients who are negative in terms of the PQ-16 or according to CHR-P criteria would be referred to the original source. For CHR-P + patients, a pretest calculator should be applied to determine whether they require specialized treatment in the early intervention program. CHR-P at low risk based on the risk calculator will be referred back to the original source with a specific treatment recommendation. All the patients with an ARMS will be assessed using a multiaxial assessment battery with specific follow-ups to monitor the possible conversion to psychosis and to update the team in charge concerning the treatment recommendations (for more details see Table A1, Appendix A).

PsyYoung introduces a consensus-based standard of referral and assessment procedures across all participating services (see Figure 1), which adopts a stepped-care approach (see Figure 2). Figure 1 describes the collaborative work between the six PsyYoung sites and project committee (in pink). Managers of PsyYoung sites are members of the steering group, and research assistants and case-managers are part of the project team. All actively participate in all of the work packages and collaborate with the institutions and patients/their relatives.

Figure 2 describes the patient referral process between the referrers (usual care settings for adolescents and young adults) and the EIS. As a first step, all potential referrals are screened with a self-rating questionnaire (prodromal questionnaire, short version; PQ-16 [20,21]) by their therapist (referrers). Based on previous studies, a PQ-16 score of 6 has been defined as the cut-off for initiating specialized psychosis risk assessment, using the SIPS or CAARMS interview (depending on the service) [22]. If the PQ-16 score is between 4 and 6, a telephone interview is conducted with the referring professional to clarify the background of the request and decide whether the psychosis risk assessment needs to be carried out. This method is employed to diminish the risk of false negatives for borderline cases at the threshold of ≤6.

In ARMS-P patients (i.e., those who meet the ARMS criteria based on the SIPS or CAARMS), a “risk-calculator” is applied to define the intensity of follow-up and treatment. The risk calculator is based on a machine-learning model of pretest risk, which was trained on historical data of referrals to early intervention services in Switzerland (*n* = 513) and achieved moderate discrimination (Harrell’s C = 0.68) (manuscript in preparation). The model stratifies patients into risk groups to help allocate clinical resources based on sociodemographic variables such as age, sex, ethnicity, and marital status, and referral source (in a manner analogous to a model described by Fusar-Poli et al., 2016 [23]). Patients at a high risk or with a FEP are recommended to receive multidisciplinary management by the intervention service according to the classical case-management approach, corresponding to the early intervention recommendations [24]. Low-risk patients are referred back to their original therapist or caregiver, to receive regular follow-up risk assessments from the early intervention service.

All ARMS-P patients, independent of risk group, additionally undergo a multiaxial assessment (Bailey et al., manuscript in preparation) to inform treatment recommendations (see Table A1, Appendix A).

With this stepped-care approach, the referral process is better defined so that referrers can carry out pre-screening and direct their patients to the EIS. Decisions concerning specialized intervention are also more precise concerning the intensity of care.

### 2.2. Measures to Increase Awareness, Dissemination and Networking

In order to increase the awareness of an ARMS, a project-dedicated Swiss-wide website (https://psyyoung.ch/en/home/ accessed on 1 May 2023) was developed in autumn 2021. This website provides information regarding the project itself (aims, sponsors, progress of the project, news), and general information to target two different populations: (a) young people and their relatives, and (b) health care and education professionals. For young people and their relatives, the site provides information on psychotic disorders, early warning signs, and access to support. For professionals, a password-protected educational platform provides information regarding ARMS diagnosis and treatment, including access to training sessions and seminars.

Moreover, specialized training sessions concerning the concepts of early detection and intervention are provided locally at regular intervals. The training sessions target potential referring professionals (‘gatekeepers’) from the educational or health care sectors, and focus on raising awareness of an ARMS, and train participants on warning signs and symptoms, as well as basic risk assessment concepts such as sensitivity and specificity. 

Finally, to improve collaboration among early intervention services in different cantons and with healthcare stakeholders, the PsyYoung consortium organizes regular networking events. These are initially with the cantonal PsyYoung project teams. Then, in the second phase, with the Swiss early intervention teams. In addition, public conferences are organized every two years at a different location. The first was in Lausanne in December 2021; the second will be in Geneva in November 2023.

### 2.3. Project Population

Regarding PsyYoung outcomes, the following populations are of interest:

(A) Patients referred to an early intervention site with a suspected clinical ARMS or FEP (expected *n* = 625, patients with a FEP are expected to constitute approximately 25% of all referred patients). Inclusion criteria: (i) age 15–35 years; (ii) at least one appointment with a specialized early intervention service in one of the three participating cantons; (iii) reason for referral being a suspected ARMS or a FEP. Participation of a close relative (see B) is not an inclusion criterion for participation of patients in the study.
(B) Close relatives of patients with a suspected ARMS or a FEP (expected *n* = 625). Inclusion criteria: (i) blood relatives or partners of patients meeting the above inclusion criteria, who (ii) live in a common household with the patient or have a close relationship (defined as contact at least once per week).
(C) Professionals involved in the detection and treatment of mental health disorders in adolescents and young adults (expected *n* = 550). Inclusion criteria: (i) professionals from one of the following groups: general practitioners/family physicians/pediatricians in the private sector/adult or child and adolescent psychiatrists or psychotherapists in the private or public sector/social workers/psychologists/other therapists in the educational sector (schools and universities, vocational consultation services etc.).

For the current study a person with an ARMS is defined as presenting: (i) attenuated positive psychotic symptoms (APS); (ii) brief and intermittent psychotic symptoms (BLIPS); or (iii) either a genetic risk (1st degree relative) or schizotypal personality disorder with a 30% drop in functioning (DRD) [5], according to the CAARMS or SIPS interviews. A FEP is defined as a person presenting a first episode of psychosis according to the CAARMS or SIPS thresholds. Patients’ psychopathology should not be explained by an organic underlying cause, or by the acute effect of substances. 

### 2.4. Study Design

**Figure 3:**
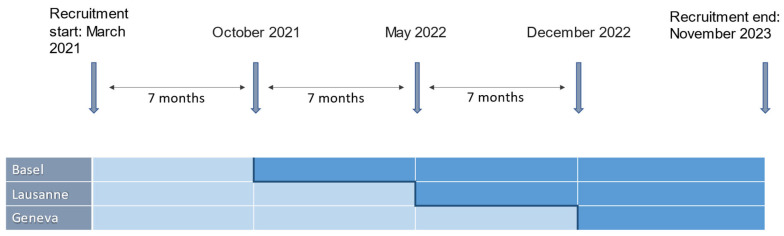
Schematic representation of the stepped-wedge study design: timeline for the implementation among PsyYoung sites. Light and dark blue indicate control and implementation phases, respectively.

The current project is an observational prospective study with a multicenter set-up. Effects of the intervention are assessed with a stepped-wedge design, a novel research study design that is increasingly being used in the evaluation of service-delivery-type interventions [25]. This type of design involves the sequential crossover of implementation sites (called “clusters”) from “control” to “intervention” in a randomized order, until all clusters are exposed to the intervention (see Figure 3 and details on statistical analyses). 

In this way, each cluster provides an unexposed group (control group, that is, the population before exposure to the intervention) and an exposed group (population after the “intervention”). This design thus involves a real-life pragmatic study design, taking into account the robust assessments and logistical constraints during the implementation of complex service delivery changes [25].

### 2.5. Setting and Procedure

Data collection started in March 2021 and will end in November 2024. The cross-over to implementation phase takes place in 7-month intervals. The order in which the cantons implement the intervention is randomized and communicated as late as practically feasible, to avoid design contamination. 

The study has been approved by all local ethics committees. Patients will sign a written general consent for the use of their clinical data (described above).

An external institute (ZHAW, Zurich University of Applied Sciences) will conduct a parallel, independent evaluation of the study. This includes questionnaires and qualitative interviews on the satisfaction with the service of patients, relatives, and referring physicians. For participation in these additional assessments, patients and relatives will sign a separate consent form and receive monetary compensation in the form of shopping vouchers (20 CHF for patients, 50 CHF for relatives); referring physicians will also sign a written consent form, and are reimbursed in the form of informative material.

### 2.6. Outcomes and Measures

The primary outcome is the number of referrals of patients with an ARMS to specialized EIS in the three participating cantons. This is assessed based on anonymized service statistics provided by each EIS at quarterly intervals.

**Figure 4:**
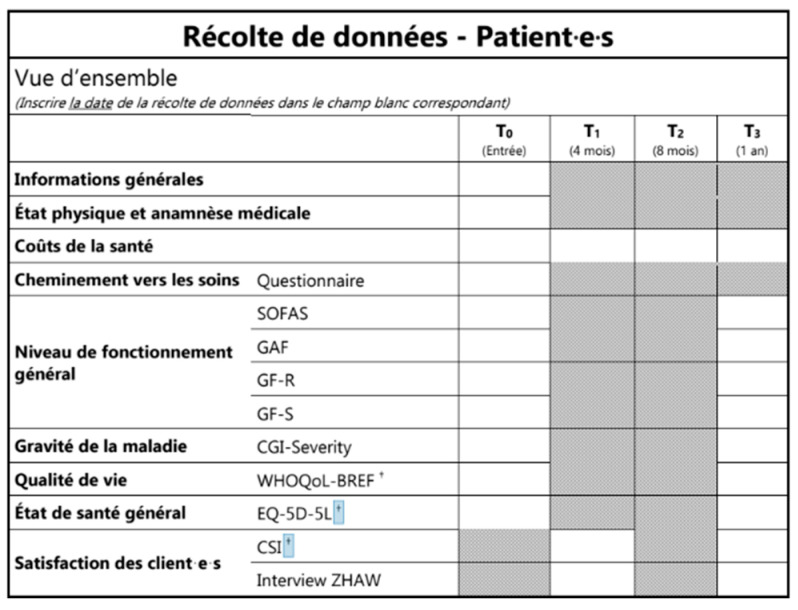
Data collection with timepoints. This table represents the first page of the CRF. The scales to be administered to the patients and the time point. The date of the assessment must be entered in the white boxes. The other boxes are greyed out because the questionnaires were not assessed at this time point. The “†” represented the self-reported assessment.

**Figure A1:**
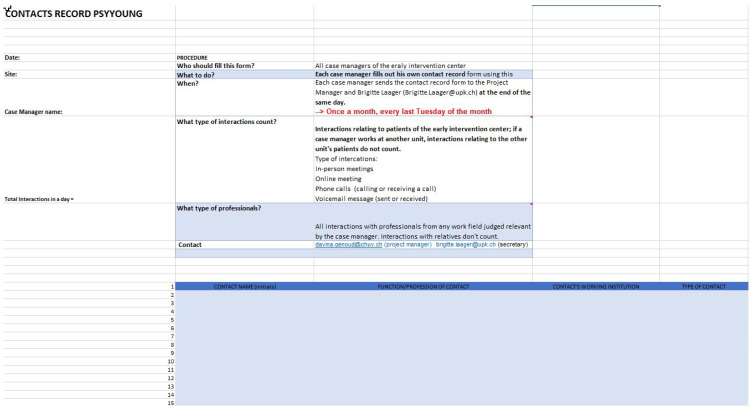
Contact monitoring.

The secondary outcomes include the following, reported on the case report form (CRF) (Figure 4):

1. Percentage of “late” referrals (i.e., referral to the EIS after a first inpatient admission). Percentage of referred patients with a suspected ARMS who are already being treated with antipsychotics at referral (contrary to current guidelines [18], and hence, a measure of insufficient awareness among referring physicians).
2. Pathway to care until the specialized referral of patients including: number of contacts with mental health professionals up until the specialized referral, delay in seeking help, delay in first referral to mental health services, and delay in referral within mental health services (see Appendix B, Table A2 and Figure A1).
3. Time from referral to needs-based orientation of referred patients. This is assessed by case managers by means of a concordance index (number of domains covered by the treatment plan divided by the number of domains identified by multiaxial needs assessment at the intake interview), based on all of the available data that is on file 1 year after the intake interview.
4. Percentage of patients who receive an individualized treatment plan within a month from first assessment.
5. Change in quality of life, functioning, and symptom severity in the first year following referral to the EIS. Quality of life is assessed by means of EuroQol 5 Dimensions (EQ-5D [26,27]) and EQ-5D-Y [28] for adults and adolescents, respectively, as well as the World Health Organization Quality of Life-BREF (WHOQOL-BREF [29,30]); measures of functioning include the functioning assessment scale (SOFAS [31]), global assessment of functioning (GAF [32]), and global functioning role and social scales (GF-R/GF-S [33]); symptom severity is assessed by the clinical global impression scale (CGI [34]).
6. Direct healthcare costs and indirect costs for patients in the first year following referral to the EIS. The assessment considers the type and dose of medication, days of absence at work or school due to illness, as well as the number of medical appointments (any specialty; divided by psychiatric, surgical, and non-surgical appointments), emergency room visits, psychotherapy sessions, other therapy sessions, and the number and duration of inpatient admissions (classified as psychiatric and non-psychiatric).
7. Duration of untreated psychosis in referred patients with a FEP.
8. Satisfaction with the EIS and the treatment plan of referred patients. This is assessed with the CSI [35], as well as qualitative analysis of semi-structured telephone interviews conducted in a subset of patients (*n* = 300) by the external evaluation institute
9. Level of engagement of patients with a ARMS within the EIS (rate of missed appointments, drop-out rate).
10. Satisfaction with the EIS of referring institutions and referring health care professionals. Opinions and evaluations from referring professionals are collected by the external evaluation institute through telephone interviews or online surveys, six months after the implementation of the intervention program.
11. Satisfaction with the EIS of the relatives of the patients. This is assessed using information collected during focus groups, conducted by the external evaluation institute in a subset of relatives (*n* = 30).
12. Quality of life and burden of care in patients’ relatives. This is assessed by means of self-reported questionnaires regarding quality of life (EQ-5D, see above) as well as the involvement evaluation questionnaire (IEQ) [36], assessing various dimensions of caregiver burden such as tension, supervision, worrying, and urging.
13. Number of interactions between specialized early intervention staff and other important players involved in the treatment and education of young people (e.g., general practitioners/pediatricians, psychiatrists and psychologists, social workers, and mental health professionals in schools, workplaces etc.). This is assessed using documentation of all interactions with the EIS clinical staff during one week at quarterly intervals (see Appendix B, Figure A1).

### 2.7. Statistics and Power Calculation

The primary endpoint (number of referrals per month) will be analyzed by Poisson regression with the cantons and the intervention as fixed effects, and time (in months) as a random effect.

Extensive simulations (10,000 replications) were performed using this generalized linear mixed model with the intercept and the variance of the month effect estimated from the monthly numbers of referrals in Basel-Stadt in 2018 and 2019. These simulations were performed assuming that the intervention effect will result in a 25% increase in the expected number of referrals, and that the expected number of referrals for the larger cantons of Vaud and Geneva will be 50% larger than for Basel-Stadt. With these assumptions and the standard significance level of 5%, the power for showing a significant effect of the intervention was 70% and the expected number of referred patients was 625.

The secondary endpoints will be analyzed using suitable generalized linear mixed models, mixed effects ordinal logistic models, and mixed-effects Cox regression models (as appropriate, depending on the variable), with at least the same fixed and random effects as for the primary endpoint.

The sample size for assessing secondary endpoints concerning relatives was estimated based on the above statistics used for estimating the patient sample size. For the secondary endpoints concerning healthcare and education professionals, the target sample size will be based on the statistics of the Swiss medical association (FMH; medical professionals), local psychotherapy registers (psychological psychotherapists), and the federal statistical office (personnel in the education sector).

## 3. Current Implementation Status and Preliminary Results

An online survey conducted in participating EIS across all PsyYoung sites (Basel-Stadt, Geneva, and Vaud) was conducted in June 2020, assessing the activities of the services in the previous 12 months. The aim of this survey was to document service characteristics such as assessment tools used at each site, duration of follow-ups for patients with an FEP and ARMS, and pathways to care. Based on this survey (manuscript in preparation), the instruments and pathway to care strategies to be applied in the implementation phase were harmonized, and the PsyYoung intervention (Figure 2) was tailored to the local context and needs.

Additionally, in the first 6 months of this project, 158 patients were referred to PsyYoung EIS (CHR: 23.4%, FEP: 31%, none: 35.4%), and 42.40% were referred after a hospitalization and did not necessarily have access to an early detection. We do not currently have enough hindsight to evaluate this effect as the implementation in Geneva started in December 2022, but with the awareness raising and training sessions, the number of referred CHR in the EIS should increase and the number of referrals after hospitalization should decrease.

Training courses have been developed collaboratively. The content of the training sessions was created collaboratively with all PsyYoung sites and is updated regularly based on participants’ feedback. 

Currently, all cantons have already crossed over to the implementation phase in 7-month intervals, starting from October 2021. In Basel-Stadt (the first canton that crossed over to implementation), three training courses were completed, including approximatively thirty participants per session; a fourth session is planned in November 2023. Lausanne EIS conducted two training courses including 40 and 34 participants, respectively; a further two courses are planned in October and November 2023. Finally, Geneva EIS have planned four training sessions starting from September 2023, to include 30 participants for each session.

**Figure 5:**
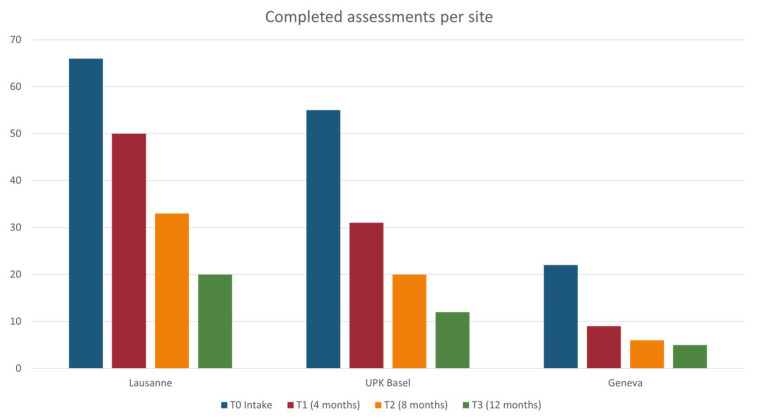
Recruitment and follow-up data (March 2023). T0 refers to baseline; T1 to 4-month follow-up, T2 to 8-month follow-up, and T4 to 12-month follow-up. The bars represent absolute numbers of participants for each site and consider both drop-outs and outstanding appointments, hence they do not represent attrition rates.

Regarding recruitment, this started in March 2021 and will continue through November 2023. Data collection is ongoing until November 2024. By March 2023, there were a total of 619 referrals to the EIS (28% FEP, 19% ARMS, 29% neither, 24% assessment not yet completed), providing anonymized data for the primary outcome as well as the secondary outcomes (i), (ii), and (x). A total of 163 patients (37% FEP, 31% ARMS patients, 32% neither) provided general signed consent for the use of their clinical data and provided data for the secondary outcomes (iii) to (ix). Of these, 26 responded to the call for a telephone interview by the external evaluation body. A total of 15 relatives consented to participate in evaluation (outcomes xii and xii). Figure 5 illustrates the number of participants assessed per center at T0 (baseline), T1 (month 4), T2 (month 8), and T3 (month 12).

## 4. Discussion

This is a transcantonal collaborative project, addressing access to and standards of early intervention for emerging psychosis in Switzerland. The project has allowed a close and fruitful collaboration between different sites across Switzerland, creating an opportunity for boosting early detection and intervention in this country. Once data collection is completed, analyses will be conducted comparing the various outcomes before and after the implementation phase. We expect that the enhanced awareness and education of stakeholders following the specific training sessions at the different sites will lead to an increase in ARMS referrals, and a reduction in late referrals and treatment with antipsychotics before referral in patients with a suspected ARMS. The pace of recruitment of patients is satisfactory, although more effort to recruit relatives is needed. The obstacles to this process are being investigated and addressed.

Given that data collection will be completed in November 2024, the first results of the project are expected to be published in 2025. We hope that these results will allow a better understanding of the current pathways to care for an ARMS and FEP; a harmonization of pathways to care for patients with an ARMS and FEP that can be applied across Switzerland; and overall, an improvement in the timely identification of an ARMS and FEP in Switzerland. In combination with health cost data and networking activities, the project is expected to provide a basis for a nationwide early intervention standard, as well as new policy measures and funding models to promote health care in young patients with emerging psychotic disorders.

A potential limitation to highlight for the clustered stepped-wedge design is ‘cross-cluster contamination’, whereby temporal trends that occur across clusters due to effects other than the intervention (e.g., increased awareness of a health issue) may reduce the statistical power [25]. In order to avoid contamination, only the heads of services were involved in preparations for project implementation and standard operating procedures. Local teams were informed regarding implementation requirements as late as practically feasible. Only after this timepoint were referral procedures and other standard operating procedures adapted to the local circumstances. Moreover, professional resources on the PsyYoung website were password-protected and only available to interested professionals in cantons that had started implementation. Similarly, the sharing of training materials with participants of the training courses in Canton Vaud took place as late as possible, approximately one month before the start of implementation in Geneva.

## Data Availability

The data collected within the project PsyYoung are available on request from the senior author. The data are not publicly available due to reasons of privacy.

## Appendix A

**Table A1:** Multiaxial assessment.

Instrument	Type and Aims
**Psychiatric Diagnosis (on Structured Level)**	
International Classification of Diseases (ICD-10/11) or Diagnostic and Statistical Manual of Mental Disorders (DSM-5)	Codified medical classification of diseases [37] Classification manual of diagnosis of mental disorders [32]
Mini international neuropsychiatric Interview (M.I.N.I.)	Short, structured diagnostic interview to assess psychiatric diagnosis according to DSM-5 and ICD-10 criteria [38]
Kiddie Schedule for Affective Disorders and Schizophrenia (K-SADS)	Semi-structured interview used to measure current and past symptoms of mood, anxiety, psychotic, and disruptive behavior disorders in children and adolescents [39]
The Structured Clinical Interview for DSM-5 Personality Disorders (SCID PD) The Structured Clinical Interview for DSM-5, Clinician Version (SCID CV)	Semi-structured diagnostic interview that guides assessment of the defining DSM-5 alternative model for personality disorders components [40] Structured clinical interview to assess DSM-5 disorders [41]
**Psychotic Symptoms**	
Positive and Negative Syndrome Scale (PANSS)	Clinical scale measuring the severity of positive and negative symptoms in patients with schizophrenia [42]
The Brief Psychiatric Rating Scale (BPRS)	Brief interview used to measure psychiatric symptoms such as anxiety, depression, and psychoses [43]
Scale for the Assessment of Negative Symptoms (SANS)	Rating scale used to measure negative symptoms in schizophrenia [44]
**Depression/Mania**	
Montgomery–Asberg Depression Rating Scale (MADRS)	Clinical scale used to assess the severity of depression in patients with mood disorders and to measure the changes brought about by the treatment of depression [45]
Beck Depression Inventory (BDI-II)	Self-administered questionnaire providing a quantitative estimation of the intensity of depressive feelings [46]
Children’s Depression Rating Scale (CDRS)	Semi-structured interview for the diagnosis of depressive disorders in children and adolescents [47]
Children’s Depression Inventory (CDI)	Self-report assessment measuring the cognitive, affective, and behavioral signs of depression [48]
Young Mania Rating Scale (YMRS)	Clinical interview scale used to assess the severity of manic states [49]
**Anxiety Disorders**	
Beck Anxiety Inventory (BAI)	Self-administered questionnaire assessing anxiety [50]
Revised Children’s Manifest Anxiety Scale (RCMAS) or Youth Anxiety Measure for DSM-5 (YAM-5)	Self-administered questionnaire measuring the level and nature of anxiety as experienced by children and adolescents [51] Self-report, or parent-report, assessing the full spectrum of symptoms of anxiety disorders in adolescents and children [52]
Social Interaction Anxiety Scale (SIAS)	Self-report scale measuring distress when meeting and talking with others [53]
Youth Self-Report for Ages 6-18 (YSR) Child Behavior Checklist for Ages 6-18 (CBCL)	Self-report questionnaire measuring emotional and behavioral problems [54] Parent questionnaire measuring behavioral and emotional problems [55]
Adult Self-Report (ASR)	Self-report questionnaire measuring emotional and behavioral problems [56]
Adult Behavior Checklist (ABCL)	Proxy Informant Questionnaire assessing psychopathology [57]
Liebowitz Social Anxiety Scale (LSAS)	Clinician-administered rating scale measuring the range of social interactions and performance situations that individuals with social phobia may fear and/or avoid [58]
Overall Anxiety Severity Impairment Scale (OASIS)	Self-reported questionnaire measuring frequency and severity of anxiety [59]
**OCD**	
Yale–Brown Obsessive Compulsive Scale (Y-BOCS)	Semi-structured interview assessing severity and frequency of obsessions and compulsions [60]
**Substance abuse, cannabis abuse**	
Alcohol, Smoking, and Substance Involvement Screening Test (ASSIST) Alcohol Use Disorders Identification Test (AUDIT) (Alternative to SCID Section)	Self-reported questionnaire for hazardous and harmful alcohol consumption [61]
Cannabis Use Disorders Identification Test (CUDIT, alternative to SCID Section)	Self-reported questionnaire for identifying cannabis use disorder in at-risk populations [62]
**Traumatic and Relevant Life Experiences**	
Childhood Trauma Questionnaire (CTQ)	Self-reported questionnaire that aims to detect experiences of childhood abuse and neglect in adults and adolescents [63]
Coddington life events scale (CLES)	Self-reported questionnaire measuring the presence and impact of important life events [64]
Perceived stress reactivity scale related to life events (PSRS)	Self-reported questionnaire measuring stress reactivity [65]
Forms of Bullying Scale (FBS)	Self-reported questionnaire assessing involvement in different forms of both bullying victimization and perpetration [66]
**Suicide Risk, Ideation**	
Risk-Urgence-Dangerosité (RUD)	Semi-structural interview evaluating the dimensions of risk, urgency and dangerosity of a suicidal patient [67]
**Other problems**	
Birchwood Insight Scale (BIS)	Self-report questionnaire measuring awareness of psychosis [68]
Paradox of Self-Stigma Scale (Pass-24)	Self-report questionnaire measuring self-stigma and related constructs (stereotype endorsement, righteous anger, and non-disclosure) [69]
Reflective functioning questionnaire (RFQ)	Self-reported measure for mentalizing [70]
Parental reflective functioning questionnaire (PRFQ-A, 12–18yrs.)	Self-reported questionnaire that assesses parental reflective functioning or mentalizing, that is, the capacity to treat the infant as a psychological agent [71]
Social Cognition Screening Questionnaire (SCSQ)	Vignette-based questionnaire used to screen for neurocognitive deficits and the patient’s needs for social cognitive intervention [72]
Multidimensional Scale of Perceived Social Support (MSPSS)	Self-report questionnaire to assess perceived adequacy of social support from family, friends, and significant others [73]
Resilience Scale for Adults (RSA)	Self-report questionnaire used to examine intrapersonal and interpersonal protective factors presumed to facilitate adaptation to psychosocial adversities [74]
**Personality disorders**	
NEO Five-Factor-Inventory (NEO-FFI)	Self-report questionnaire that provides a measure of the five personality domains [75]
Level of Personality Functioning Questionnaire (LoPF-Q 12-18)	Self-report questionnaire measuring alterations in the functional level of personality in four areas [76,77]
**Autism**	
Social Communication Questionnaire (SCQ)	Self-report questionnaire to screen and monitor communications skills and social functioning in children who may have autism/autism spectrum disorders [78]
**TDAH**	
Conners Adult ADHD Rating Scales (CAARS) Conners Comprehensive Behavior Rating Scales (Conners CBRS)	Self-report, or observer report, to assess, diagnose and monitor treatment of ADHD in adults [79] Self-report questionnaire used to diagnose attention deficit disorder with or without hyperactivity [80]
Wender Utah Rating Scale (WURS, adults)	Self-report questionnaire used in the diagnosis of attention deficit hyperactivity disorder based on behavior and feelings experienced during childhood [81]
**Neurocognition**	
Measurement and Treatment Research to Improve Cognition in Schizophrenia (MATRICS)	Self-report questionnaire measuring cognition in individuals diagnosed with schizophrenia and related disorders [82]
Brief Assessment of Cognition in Schizophrenia (BACS)	Test battery assessing aspects of cognition found to be the most impaired in patients with schizophrenia [83]
A developmental NEuroPSYchological Assessment (NEPSY)	Self-report questionnaire that is tailored assessment of child and adolescent skills in 6 major neuropsychological areas [84]
**Somatic Assessment**	
Recommendations: SGPP Behandlungsempfehlungen Schizophrenie	Recommendations for the treatment of schizophrenia [85]
The exams listed are for the exclusion of organic psychosis	For patients with psychosis, our recommendations are as follows; For patients *at-risk* of psychosis, the exams depend on the specific situation and specific clinical concerns.
Complete somatic assessment	
Complete blood cell (CBC) count	
Electrolyte (incl. Calcium) levels	
CRP	
Liver and renal function test	
TSH	
Drug Screening	
Vitamin B-12	

## Appendix B

**Table A2:** Pathway to care in the CRF.

Cheminement Vers les Soins						
CONTACTS *(du Premier Contact [1] au Dernier [x]; Inscrire Uniquement les Chiffres/Lettres de la Légende)*						
	Date	Contact Initié Par *(Légende A–D)*	Qui a Été Contacté *(Légende E–R)*	Bon Contact *(Oui/Non)*	Raisons de la Prise de Contact *(Légende 1–12)*	Propositions à L’issu du Contact *(Légende 13–18; Entourer les Propositions Suivies)*
1	__ __/__ __/__ __ __ __					
2	__ __/__ __/__ __ __ __					
3	__ __/__ __/__ __ __ __					
4	__ __/__ __/__ __ __ __					
5	__ __/__ __/__ __ __ __					
6	__ __/__ __/__ __ __ __					
7	__ __/__ __/__ __ __ __					
8	__ __/__ __/__ __ __ __					
9	__ __/__ __/__ __ __ __					
**LÉGENDE**						
*Contact Initié Par*			*Qui a Été Contacté*		*Raisons de la Prise de Contact*	*Propositions*
A. patient·e seul·e B. famille seule C. patient·e + famille D. autre (*préciser*)			E. médecin traitant·e F. pédopsychiatrie/psychiatre privé G. pédopsychiatre/psychiatre public H. psychologue I. service d’urgence d’un hôpital général J. hôpital psychiatrique K. infirmier/infirmière scolaire L. enseignant·e M. éducateur/éducatrice N. travailleur/travailleuse social·e O. police P. clergé Q. guérisseur/guérisseuse R. autres		Symptômes psychotiques tristesse ou déprime stress ou anxiété confusion ou désorganisation colère ou irritabilité retrait social détérioration des soins d’hygiène troubles du sommeil fatigue ou épuisement risque suicidaire autres inconnues	13. de référer à un·e pédiatre/généraliste 14. de référer à un·e pédopsychiatre/psychiatre 15. d’adresser à un hôpital psychiatrique 16. de référer à une unité ou plateforme spécialisée en CHR-P/FEP 17. d’introduire un traitement psychotrope: a. lequel (préciser: AD/BZD/AP) et dosage b. compliance: i. nulle (<25%) ii. partielle (25–75%) iii. complète (>75%) 18. autres
**NOMBRE DE CONTACTS**				Durant la phase prodromique: _____ Durant la phase psychotique: _____ Indissociable: _____ **total**: _____		
